# Does ivermectin have a place in the treatment of mild Covid-19?

**DOI:** 10.1016/j.nmni.2022.100985

**Published:** 2022-05-27

**Authors:** Eli Schwartz

**Affiliations:** The Center for Geographic Medicine and Tropical Diseases, The Chaim Sheba Medical Center, Tel Hashomer & Sackler Faculty of Medicine, Tel-Aviv University, Tel-Aviv, Israel

**Keywords:** Coronavirus treatment, hospitalization, molnupiravir, paxlovid, SARS-CoV-2

Ivermectin has been used to treat humans for the past 4 decades. It was approved as a broad spectrum anti-parasitic agent, initially indicated in 1987 to treat onchocerciasis and was given as a mass drug administration (MDA) in endemic countries. Its success awarded the discoverers the Nobel prize of Medicine in 2015. Ivermectin's principal activity was to treat infections caused by roundworm parasites. Over the years, the spectrum was broadened to include ectoparasites such as scabies among others. Through the years more than 3 billion doses have been given to humans (not to horses) with a high safety profile, and the drug was added to the World Health Organization's List of Essential Medicines [https://apps.who.int/iris/bitstream/handle/10665/345533/WHO-MHP-HPS-EML-2021.02-eng.pdf ].

In the last decade, several in-vitro studies have shown its anti-viral activity against a broad range of viruses. At the beginning of the COVID pandemic, ivermectin was tested in vitro against SARS-CoV-2 and showed a highly significant reduction (99.8%) in viral RNA after 48 hours [1], but it was criticized that this was achieved by using a much higher dose in comparison to the standard dose in human use [2]. However, its anti-COVID activity in real-life in patients who were treated with standard dose of 3 days of ivermectin showed the significant reduction in culture viability in the ivermectin group compared to placebo [3]. In addition, ivermectin has anti-inflammatory properties based on in-vitro and in animal model studies. An extensive review of the potential mechanisms of action for ivermectin against COVID-19 was recently published [4].

SARS-CoV-2 infection includes several stages, where the initial stage is manifested by high viral replication followed by the second stage (occurring in the high risk groups mainly) of excessive inflammatory response causing severe disease and death. Therefore, ivermectin may have a dual role in this infection, acting as both an anti-viral and anti-inflammatory agent. With its high safety profile, ivermectin is a potential treatment against COVID-19 in its different stages.

There are already over 80 studies assessing the impact of the drug in the different stages of the disease such as: preventing infection, shortening viral shedding, preventing hospitalization, and death and more [https://ivmmeta.com]. Several reviews and meta-analyses disputing the value of this drug in fighting the COVID pandemic were published with the main argument against using ivermectin being that the level of existing evidence for its positive effect is based mainly on studies lacking a high standard of rigorous methodology [5,6]. One must also consider however that since the drug no longer has rights for patent, pharmaceutical companies have no monetary incentive to conduct clinical trials using this medication, and governmental agencies are reluctant to sponsor trials for drug repurposing.

Efforts have been made to identify high quality studies in order to come to consensus [5]. In regard to reduced hospitalization for patients receiving the drug at the early stage of the disease, three studies were identified by Hill et al. [3,7,8]. Combing the results of these three studies, with the recent TOGETHER trial in Brazil [9], there has been shown a significant reduction in hospitalization, with risk ratio of 0.74 (p = 0.02) [see Fig. 1]. In all studies patients were recruited within 7 days from symptom onset (at a median of 4-5 days).

**Fig. 1:**
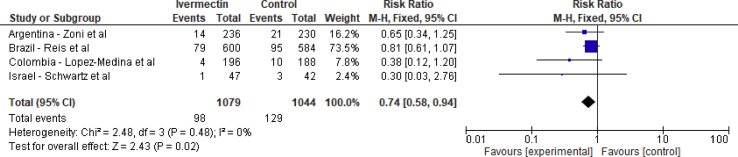
Effect of ivermectin on prevention of hospitalization including studies with low concerns for bias.(Ref [3,[7], [8], [9]]).

The introduction of COVID 19 vaccines was a game changer in fighting the pandemic. However, it has become clear that we cannot rely on vaccines as a sole agent in this battle. The phenomena of waning immunity within several months, the viral mutations which can elude the virus from vaccine efficacy, all highlight the need for anti-viral drugs to prevent deterioration, hospitalization and death. In fact even the big vaccine/pharma companies have entered the race of finding an efficient anti-COVID drug.

In addition to the repurposed-drug ivermectin mentioned above, the first new drug to show anti-COVID activity was molnupiravir (manufactured by Merck). It initially demonstrated a 50% reduction in hospitalization in high-risk groups but in final analysis, unfortunately showed only a 30% decrease in hospitalization [10].

The second new drug was nirmatrelvir, which given together with ritonavir under the brand name paxlovid (manufactured by Pfizer), showed an 89% reduction in hospitalization [11]. The main disadvantage of paxlovid is the potential and serious interactions with a long list of medications and it is also contraindicated for use in those with certain medical conditions. Unfortunately these are often the patients with co-morbidities, on a chronic medication regime who are in need of this drug. Another drawback of these two drugs is the fact that they should be administered within five days of symptom onset, and the treatment course costs several hundreds USD.

Both drugs (paxlovid and molnupiravir) were received an emergency use authorization by the FDA, and were authorized to be used by health authorities in many countries. So far there have been no published post-marketing field studies looking at the real-life results of their efficacy in preventing hospitalization, death as well as the actual profile of their adverse-events. In addition, unpublished data by Pfizer about paxlovid efficacy in mixed populations of vaccinated and non-vaccinated patients (who are the current candidates for therapy these days), showed that the drug did not reach a significant advantage over placebo [12].

## So, is there a role for ivermectin early treatment in an era when we have new anti-COVID drugs?

Comparing molnupiravir to ivermectin in preventing hospitalization [Table 1], we see that based on existing data the performance of ivermectin is similar to molnupiravir. Furthermore, ivermectin has a good safety-profile in terms of adverse events, and in addition the cost is in order of 100 times less when compared to the newer drugs. In addition, ivermectin is easily available in most countries, an important fact especially in low-middle-income countries where getting these newer and expensive drugs may not be feasible. Accordingly, in countries where the two new medications (paxlovid and molnupiravir) are not available, ivermectin should be offered.

**Table 1:** Risk of hospitalization in non-hospitalized patients treated with ivermectin vs. molnupiravir

*Drug* (Ref)	Ivermectin [3,[7], [8], [9]]	Molnupiravir [10]
Total number of participants	2346 Drug- 1079/Placebo-1044	1408 Drug-709/Placebo-699
% High Risk	Any co-morbidity = ∼75%	All
% positive in Placebo arm	129/1170 = 12.3%	68/699 = 9.7%
% positive in active drug arm	98/1176 = 9.0%	48/709 = 6.8%
Risk Ratio (95% CI)	0.74 (0.58-0.94)	0.69 (0.48 to 1.01)
P-value	0.02	0.04

Even in high-income countries where these two drugs might be available, there remains a role for ivermectin. Data from MOH in Israel show that only the minority (about 10%-15%) of high-risk patients are actually getting treatment. This is due to the limited supply of these drugs, the hesitancy to take these drugs related to fear of their unknown adverse events or to known contraindications in using them. Therefore offering them ivermectin should be a viable option.

Since in most countries ivermectin has not been approved for COVID treatment, performing ivermectin vs. placebo studies appears to be unethical when these two newer drugs have been officially approved by health-authorities. However, offering ivermectin to those who refuse the new drugs seems to be a reasonable option. Since eligibility criteria in getting these early treatments are targeted to high-risk patients only (although the definition of high risk might differ from one country to the other), observing the outcome of these arms of treatment: paxlovid vs. molnupiravir vs. ivermectin might shed light on the value of ivermectin in comparison to the newer drugs. Since, undoubtedly there will be patients who will refuse any treatment, they in fact can serve as a control arm receiving SOC (standard of care). Although COVID regulations are loosening, the pandemic is still circulating and therefore this suggested prospective observational study is still highly important and timely, and potentially will give us the value of each treatment arm.

## Transparency declaration

The Author declare no conflict of interest.

## Funding

No fundings.
