# Corrigendum to “Does ivermectin have a place in the treatment of mild Covid-19?” [New Microbes New Infect 46 (C) (2022 Mar) 100985]

**DOI:** 10.1016/j.nmni.2022.101000

**Published:** 2022-07-01

**Authors:** Eli Schwartz

**Affiliations:** The Center for Geographic Medicine and Tropical Diseases, The Chaim Sheba Medical Center, Tel Hashomer & Sackler Faculty of Medicine, Tel-Aviv University, Tel-Aviv, Israel

The author regrets having incorrectly used in the Forest-Plot figure, under the column of “Total” the numbers which were the “non-events” numbers instead of the Total numbers. This is corrected now and has changed the Risk Ratio (95% CI) being now 0.77 (0.60-0.98) and P = 0.04.

These numbers were corrected in the figure and also in the last 2 lines of Table 1.

The corrected Figure and |Table are enclosed.

These changes did not affect any of the conclusions of the manuscript.

The authors would like to apologise for any inconvenience caused.

Fig. 1. Effect of ivermectin on prevention of hospitalization including studies with low concerns for bias. (Ref [3,7–9]).

**Figure:**
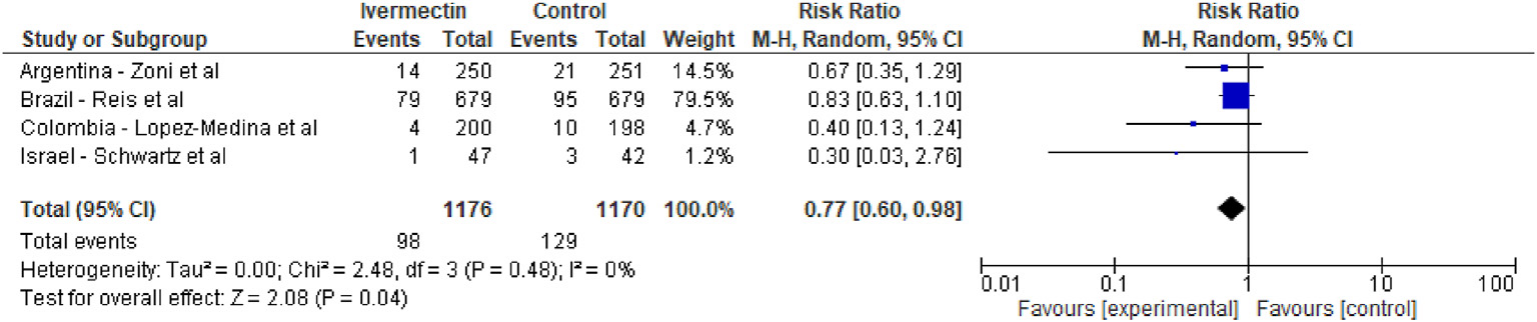

**Table:** Table 1. Risk of hospitalization in non-hospitalized patients treated with ivermectin vs. molnupiravir

*Drug* (Ref)	Ivermectin (3,7-9)	Molnupiravir (10)
Total number of participants	2346 Drug- 1079/Placebo-1044	1408 Drug-709/Placebo-699
% High Risk	Any co-morbidity = ∼75%	All
% Positive in Placebo arm	129/1170 = 12.3%	68/699 = 9.7%
% Positive in active drug arm	98/1176 = 9.0%	48/709 = 6.8%
Risk ratio (95% CI)	0.77 (0.60-0.98)	0.69 (0.48 to 1.01)
P-value	0.04	0.04

